# Transcriptional Alterations of Virulence-Associated Genes in Extended Spectrum Beta-Lactamase (ESBL)-Producing Uropathogenic *Escherichia coli* during Morphologic Transitions Induced by Ineffective Antibiotics

**DOI:** 10.3389/fmicb.2017.01058

**Published:** 2017-06-13

**Authors:** Isak Demirel, Ignacio Rangel, Ulrika Petersson, Katarina Persson, Robert Kruse

**Affiliations:** ^1^School of Medical Sciences, Örebro UniversityÖrebro, Sweden; ^2^Faculty of Medicine and Health, Inflammatory Response and Infection Susceptibility Centre, Örebro UniversityÖrebro, Sweden; ^3^Faculty of Medicine and Health, Nutrition-Gut-Brain Interactions Research Centre, Örebro UniversityÖrebro, Sweden; ^4^Department of Clinical Research Laboratory, Faculty of Medicine and Health, Örebro UniversityÖrebro, Sweden

**Keywords:** filamentation, extended-spectrum β-lactamase, uropathogenic *Escherichia coli*, ineffective antibiotics, morphological plasticity

## Abstract

It is known that an ineffective antibiotic treatment can induce morphological shifts in uropathogenic *Escherichia coli* (UPEC) but the virulence properties during these shifts remain to be studied. The present study examines changes in global gene expression patterns and in virulence factor-associated genes in an extended spectrum beta-lactamase (ESBL)-producing UPEC (ESBL019) during the morphologic transitions induced by an ineffective antibiotic and in the presence of human primary bladder epithelial cells. Microarray results showed that the different morphological states of ESBL019 had significant transcriptional alterations of a large number of genes (Transition; 7%, Filamentation; 32%, and Reverted 19% of the entities on the array). All three morphological states of ESBL019 were associated with a decreased energy metabolism, altered iron acquisition systems and altered adhesion expression. In addition, genes associated with LPS synthesis and bacterial motility was also altered in all the morphological states. Furthermore, the transition state induced a significantly higher release of TNF-α from bladder epithelial cells compared to all other morphologies, while the reverted state was unable to induce TNF-α release. Our findings show that the morphological shifts induced by ineffective antibiotics are associated with significant transcriptional virulence alterations in ESBL-producing UPEC, which may affect survival and persistence in the urinary tract.

## Introduction

Urinary tract infections (UTIs) are one of the most common bacterial infections. UTI is especially common in women as nearly half of all women will be infected during their lifetime (Foxman, 2003). The vast majority of all urinary tract infections are caused by uropathogenic *Escherichia coli* (UPEC), accounting for approximately 80% of community-acquired symptomatic UTI and approximately 25% of the nosocomial infections (Ronald, 2003). UPEC isolates have been shown to express an array of virulence factors like adhesins (such as P- and type-1 fimbriae), toxins (α-hemolysin), lipopolysaccharide (LPS), capsular, siderophores (iron scavenger system) and TcpC to evade the host defenses and colonize the urinary tract (Bower et al., 2005; Yadav et al., 2010).

During UTI, UPEC may invade the urothelial cells where they can replicate and form biofilm-like intracellular bacterial communities (IBCs) (Anderson et al., 2003). Likewise, after the resolution of an active infection non-replicating bacteria can remain within vesicles in deeper layers of the urothelium as quiescent intracellular reservoirs (QIRs) (Mysorekar and Hultgren, 2006). These QIRs can remain quiescent for several months without eliciting an inflammatory response. The intracellular niche not only protects the bacteria from many of the innate immune defenses, such as phagocytosis by neutrophils (Justice et al., 2004), but also from antibiotics that often are ineffective in eliminating intracellular bacteria (Blango and Mulvey, 2010). The subsequent resurgence of these intracellular reservoirs can be the cause of recurrent or chronic UTI (Rosen et al., 2007). UPEC exhibit morphological plasticity within the IBCs (Justice et al., 2004, 2006; Horvath et al., 2011). In the initial phase, bacteria transform from a rod shape to coccoid morphology that may follow by an additional transition into a filamentous form (Justice et al., 2004; Horvath et al., 2011). As bacteria emerge from the IBC they may do so in a filamentous form (Justice et al., 2004). Filamentous *E. coli* have been observed in urine from women with UTI and the filamentation *per se* may serve as a strategy to resist host immune responses, such as phagocytosis (Horvath et al., 2011), but may also be seen as a more general stress response. Filamentation can be induced by components of an activated host defense system (Justice et al., 2006), shear stress (Khandige et al., 2016) and also in response to treatment with different beta-lactam antibiotics, in particular, aminothiazolyl cephalosporins like ceftibuten. Beta-lactam antibiotics induce *E. coli* filamentation by inhibiting penicillin-binding protein-3 (PBP-3) that catalyzes septa formation (Gould and MacKenzie, 1997). SulA, a member of the bacterial SOS DNA damage repair system, has also been shown to induce bacterial filamentation. It was recently shown that cell division gene *damX* is also a mediator of reversible filamentation of UPEC (Khandige et al., 2016). We have previously shown that filamentation of extended spectrum β-lactamase (ESBL)-producing UPEC isolates can alter the ability of UPEC to evoke pro-inflammatory responses in renal epithelial cells and neutrophils (Demirel et al., 2015). Therefore, knowledge of the virulence factors expressed by different morphological forms of UPEC may be used to devise novel therapeutic strategies to obstruct these virulence factors in order to inhibit pathogenesis.

A major concern and emerging health problem is the increasing incidence of antibiotic resistance among uropathogenic strains (Coque et al., 2008; Brolund, 2014). A majority of the ESBL-producing bacteria are isolated from urine samples (Pitout and Laupland, 2008; Khanfar et al., 2009). In addition, mortality has been shown to increase in ESBL-induced infections proportionally with the duration of the delay in effective treatment, making the choice of correct antibiotics critical for patients (Tumbarello et al., 2007).

The virulence capacity of ESBL-producing UPECs during their morphological shifts remains to be properly studied. The aim of the present study was to investigate changes in global gene expression patterns and in virulence factor-associated genes in ESBL-producing UPEC during the morphologic transitions induced by ineffective antibiotics in the presence of human primary bladder epithelial cells.

## Material and methods

### Cell and bacterial culture

Primary human bladder epithelium progenitor cells (HBEP, CELLnTEC Advanced Cell Systems AG, Bern, Switzerland) were cultured in wells with CnT-58 cell culture medium (CELLnTEC) supplemented with 100 U/mL penicillin and 100 μL/mg streptomycin (both from Invitrogen Ltd, Paisley, UK) in a humidified atmosphere with 5% CO_2_ at 37°C. At confluency the culture was differentiated during 4 days using CnT-21 differentiation medium (CELLnTEC) supplemented with 1 mM CaCl_2_. The ESBL-producing UPEC was originally isolated from a patient at Örebro University hospital, Sweden. The isolate, designated ESBL019 (previously named ESBL7, Demirel et al., 2015), has previously been characterized and determined to be multidrug resistant (CTX-M-15) and resistant to ceftibuten (MIC 7,680 ng/ml) and belongs to the ST131 clone (Onnberg et al., 2011). ESBL019 was grown in Luria broth (Difco Laboratories, Detroit, MI, USA) overnight on shake at 200 rpm 37°C. The bacteria were resuspended in sterile phosphate buffered saline prior to inoculation of CnT-21 cell culture medium (CCM) with or without ceftibuten (480 ng/mL). The antibiotic concentration used is a reference minimal inhibitory concentration (MIC) of ceftibuten that was determined on two antibiotic susceptible UPEC isolates as previously defined (Demirel et al., 2015).

### Morphologic transitions and evaluation

**Figure 1:**
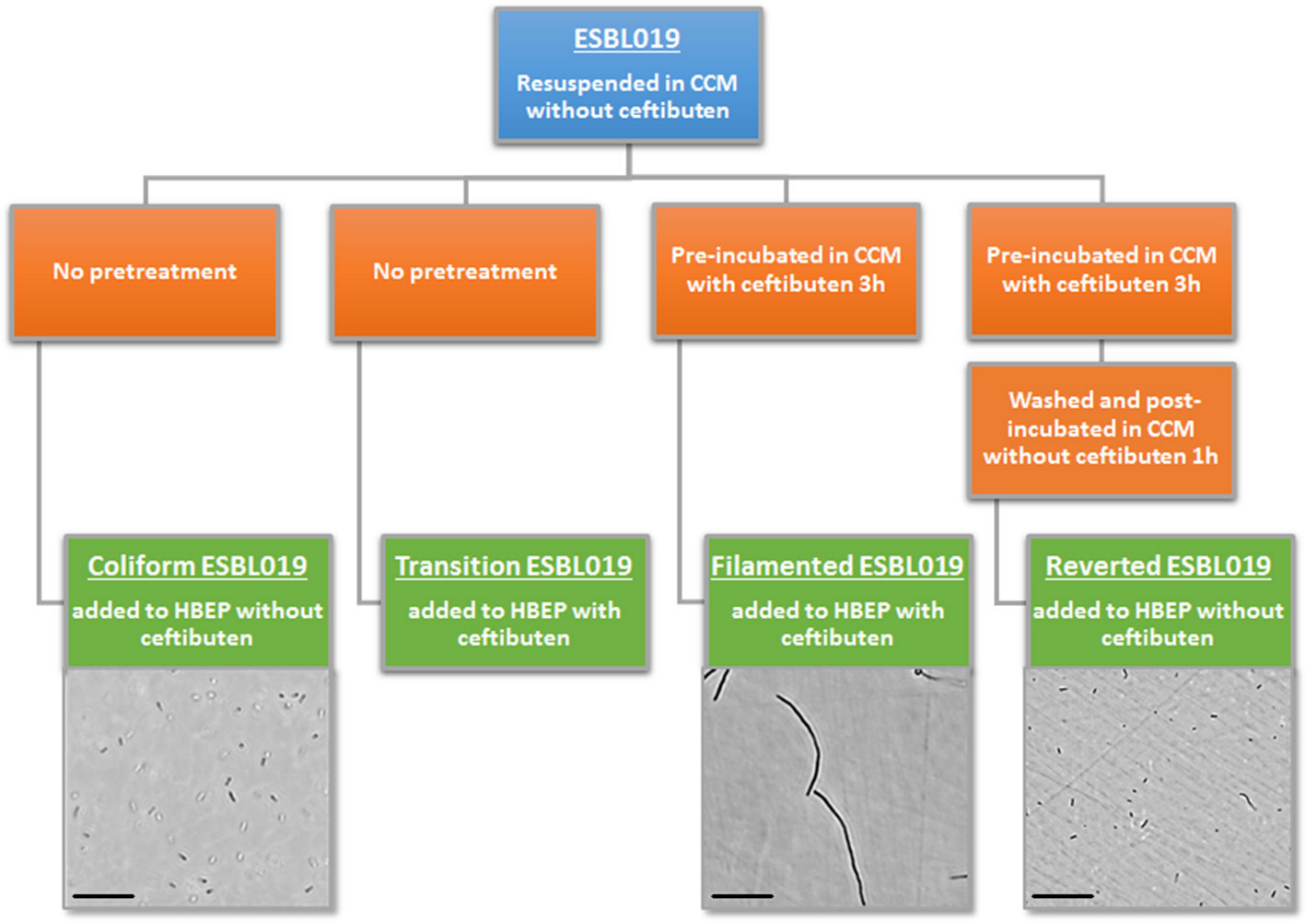
Summary of the experimental design, morphological transition and visual evaluation by light microscope of the different forms of ESBL019. Scale bar: 100 μm.

Four morphologic states of ESBL019 were used during the experiments (Figure 1). A first ESBL019 morphological state was prepared by resuspension of ESBL019 in CCM without ceftibuten supplementation prior to its inoculation of HBEP cells without ceftibuten (designated; ESBL019 Coliform). A second ESBL019 morphological state was prepared by resuspension in CCM and then used to inoculate HBEP cells with ceftibuten supplementation (480 ng/mL) (designated; ESBL019 Transition). This transition from coliform into filamented occurs in the presence of HBEP cells. A third ESBL019 morphological state was prepared by resuspension and pre-incubation for 3 h in CCM with ceftibuten in a tube, in order to induce complete filamentation of all bacteria. Filamentation was confirmed by microscopy. The third pre-filamented ESBL019 fraction was used to inoculate HBEP cells in the presence of ceftibuten to keep the bacteria filamented (designated; ESBL019 Filamented). The CCM of the pre-filamented ESBL019 fraction was replaced with CCM without ceftibuten and the filamented ESBL019 were allowed to revert completely back to its coliform during 1 h in a tube prior to the inoculation of HBEP cells without ceftibuten (designated; ESBL019 Reverted) (Figure 1). The bacterial morphologies were evaluated with an Olympus CKX41 inverted light microscope (Olympus, Segrate, Italy). Prior to inoculation the bacterial concentrations of all fractions were adjusted in order to inoculate HBEP cells with a multiplicity of infection (MOI) of 10. All inoculated cells were incubated for 4 h in 5% CO_2_ at 37°C after which supernatants were collected and centrifuged 5 min at 5,000 × g to collect the extracellular bacteria. The viability of the HBEP cells was >90% after the infection as determined by the neutral red assay. The HBEP cells were then lysed with sterile MQ water for 3 min and the intracellular bacteria were collected and centrifuged for 5 min at 5,000 × g followed by a washing step with PBS.

### RNA preparation and microarray

Total RNA was isolated from the combined intra-and extracellular bacteria with the RNeasy Mini Kit (Qiagen Technologies, Hilden, Germany) according to manufacturer instructions. Contaminating DNA was removed by DNase digestion (TURBO DNase, Life technologies, MA, USA) according to manufacturer instructions. RNA concentration and purity were determined using a Nano-Drop ND-1000 Spectrophotometer (Nano-Drop Technology Inc., Wilmington, DE, USA). All samples had OD260/280 and OD260/230 ratios above 1.9. RNA quality was further evaluated using Agilent 2100 Bioanalyzer (Agilent Technologies, Palo Alto, CA, USA) according to manufacturer instructions. All samples had RNA integrity number (RIN) values above 9. High-quality total RNA from four biological replicates was used to prepare labeled cRNA with Low Input Quick Amp WT Labeling Kits (Agilent) according to manufacturer instructions. Labeled cRNA samples were hybridized in a G2545A hybridization oven (Agilent) onto Agilent SurePrint G3 *E. coli* Gene Expression 8x15k (Agilent Technologies) glass arrays according to manufacturer instructions and subsequently scanned with a G2565 CA array laser scanner (Agilent Technologies). Image analysis and data extraction was performed with Feature Extraction Software (version 10.7.3.1, Agilent Technologies). The virulence factor list for *E. coli* was generated through the use of virulence association in the PATRIC database (www.patricbrc.org) with the addition of MESH virulence search term associated genes and literature mining. Gene expression data is available in the GEO database with the accession number GSE99661.

### Quantitative real-time PCR (qPCR)

cDNA synthesis (200 ng of total RNA) was performed by using High Capacity cDNA Reverse Transcription Kit for single-stranded cDNA synthesis (Applied Biosystems, CA, USA) according to manufacturer‘s protocol. Maxima SYBR Green qPCR Master Mix (ThermoFisher Scientific, MA, USA) was used for the qPCR according to manufacturer's instructions. Two hundred and fifty nanometer of primer and 10 ng template cDNA was added to each reaction. Primers were ordered from Eurofins MWG Synthesis GmbH (Ebersberg, Munich, Germany) (Table S1). The amplification was performed in a CFX96 Touch™ Real-Time PCR Detection System (Biorad, CA, USA) using the following protocol: initial denaturation at 95°C for 10 min, 40 cycles of denaturation at 95°C for 15 s followed by annealing at 60°C for 30 s and extension at 72°C for 30 s. The PCR was followed by a dissociation curve analysis between 60 and 95°C. The Ct values were analyzed by the comparative Ct (ΔΔCt) method and normalized to the endogenous control gapA (encoding glyceraldehyde 3-phosphate dehydrogenase A). Fold difference was calculated as 2^−ΔΔCt^.

### Transmission electron microscopy

ESBL019 was prepared for electron microscopy with or without ceftibuten as previously mentioned, but without the presence of HBEP cells. ESBL019 was washed in PBS before fixation with 5% glutaraldehyde in 0.1 M phosphate buffer. The bacteria were absorbed on carbon-coated copper grids and contrasted with 1% uranyl acetate solution. A JEOL JEM 1230 transmission electron microscope at 80-kV accelerating voltage was used for examination and images were recorded with a Gatan Multiscan 791 CCD camera.

### Agglutination assays

ESBL019 in its four different morphological states were collected after HBEP inoculation to measure Type-1-and P-fimbriae phenotype expression. The expression of Type-1 fimbriae was evaluated using 5% yeast diluted in PBS and the expression of P-fimbriae was evaluated using 2% of P-positive human erythrocytes diluted in PBS. All the bacteria from the well were mixed 1:1 (v/v) with yeast or erythrocytes on a glass slide and agglutination was evaluated with an Olympus CKX41 inverted light microscope.

### Measurement of TNF-α release from primary bladder epithelial cells

An enzyme-linked immunosorbent assay (ELISA) was performed to measure the TNF-α release from HBEP cells in response to the different morphological states of ESBL019. TNF-α was measured using the human TNF-α kit (ELISA MAX™ Standard, BioLegend, San Diego, CA, USA) according to the manufacturer's protocol and measured on a spectrophotometer (Multiskan Ascent, Thermo Labsystems, Helsingfors, Finland).

### Statistical analysis and microarray data processing

All microarray data analysis was performed using Gene Spring GX version 13.0 (Agilent Technologies) after per chip and gene 75th percentile shift normalization of samples. Different expression between groups was analyzed with one-way analysis of variance (ANOVA) parametric test. Significantly expressed entities (*p* < 0.05) was obtained by Tukey HSD *post hoc* test followed by Bonferroni multiple testing correction and a fold change set at ≥2. Significance for GO term enrichment and single experiment pathway analysis were set at a *p*-value < 0.05 and *p*-value < 0.1 respectively. The virulence factor list for *E. coli* was generated through the use of virulence association in the PATRIC database (www.patricbrc.org) with the addition of MESH virulence search term associated genes and literature mining. In total the list was composed of 305 genes classified as associated with or designated to virulence. The differences between groups in the ELISA assay were evaluated with one-way ANOVA followed by Bonferroni test. The differences compared to ESBL019 Coliform in the qPCR were evaluated with Student's unpaired *t*-test. Differences were considered statistically significant when *p* < 0.05. Data are presented as mean ± standard error of the mean (SEM), *n* = number of independent experiments.

## Results

### Morphological plasticity of *E. coli* in response to ceftibuten exposure

**Figure 2:**
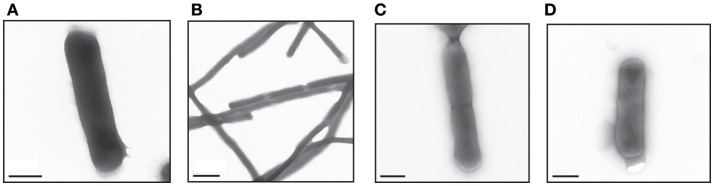
Visual evaluation of the different forms of ESBL019 by transmission electron microscope. ESBL019 Coliform **(A)** was grown for 3 h without ceftibuten, ESBL019 Filamented **(B)** was grown for 3 h in the presence of ceftibuten and ESBL019 Reverted **(C,D)** was grown in the presence of ceftibuten for the first 3 h and then without for 1 h to allowed the bacteria to revert completely back to its coliform. Septa formation during reversion can also be observed **(C)**. Scale bar: 0.5 μm **(A,C,D)** 2 μm **(B)**.

ESBL019 is a strain resistant to ceftibuten. It changed morphology from its normal coliform into a filamentous form (>100 μm) after exposure to ceftibuten in the presence of human primary bladder epithelial cells (HBEP). This transition in the presence of HBEP is defined as ESBL019 Transition. The filamentation process started as early as 1 h after the addition of ceftibuten and the bacteria reached full filamentation approximately 3 h after exposure to ceftibuten. After removing ceftibuten from the growth medium, the filamentous ESBL019 reverted completely back to its coliform within 1 h (Figure 1). Transmission electron microscopy showed a normal Coliformed bacteria (Figure 2A) and that the septa between the bacteria was not fully formed in the filamented phase (Figure 2B). The reversion from the filamentous form back to the coliform was associated with formation of the bacterial septa (Figures 2C,D).

### Gene expression alterations in the different morphological phases

**Figure 3:**
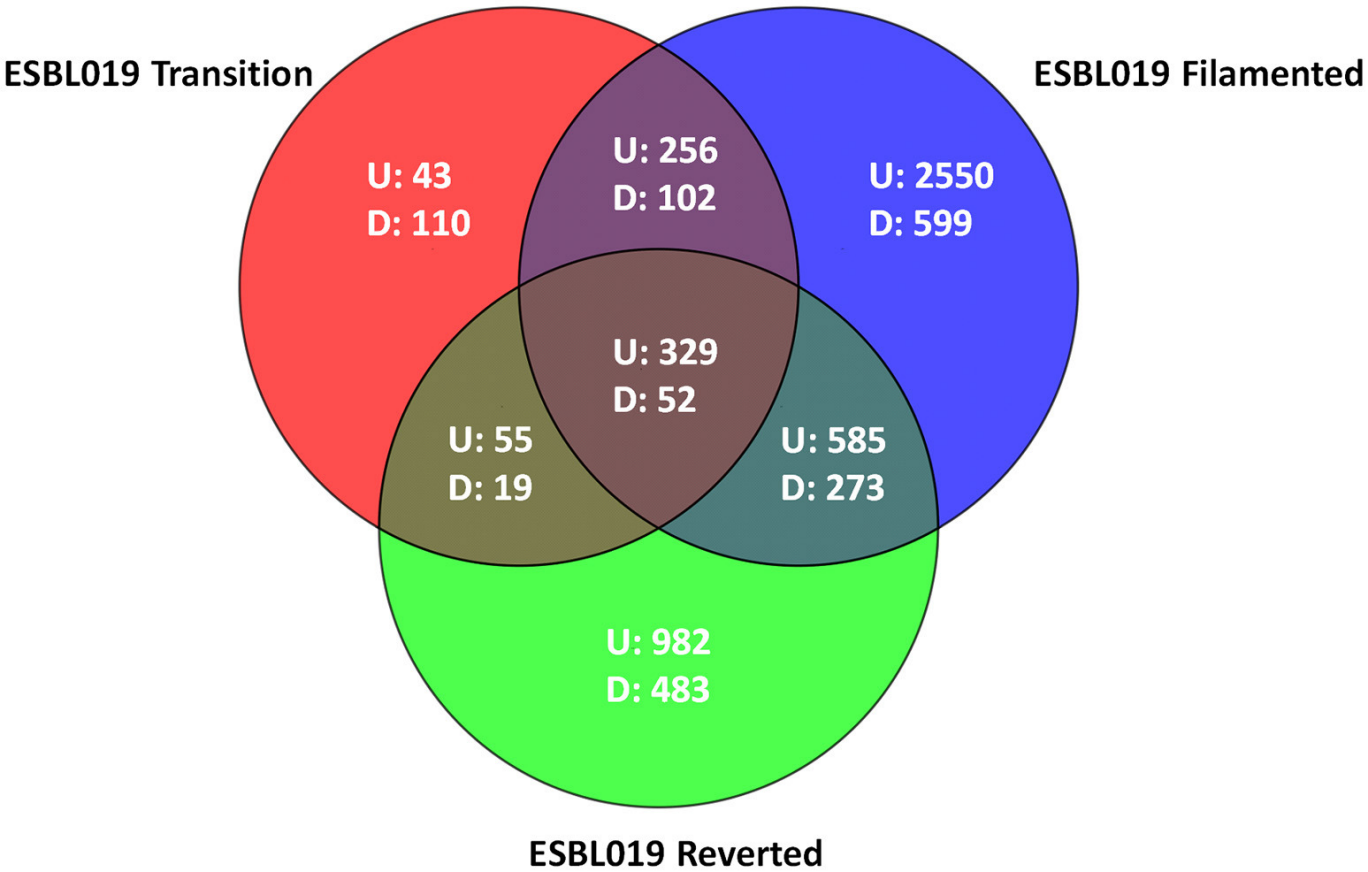
Venn diagram of differentially expresses entities. Red represents ESBL019 Transition compared to ESBL019 Coliform, blue represents E7 Filamented compared to ESBL019 Coliform and green represents ESBL019 Reverted compared to E7 Coliform. Overlapping regions represents shared entities. Up- and down-regulated entities are designated U and D respectively. *n* = 4 per group.

Microarray analysis was performed on total RNA isolated from (intra and extracellular) bacteria prior to and following their morphological transition from coliform to filamentous form and then reverted to their original coliform. In total 6438 entities were differentially expressed (*p* < 0.05) with at least a ≥2 fold change compared to the ESBL019 Coliform. All three morphological states, ESBL019 Transition, ESBL019 Filamented and ESBL019 Reverted, induced changes in gene expressions with 683, 3,720, and 1,951 up-regulated and 283, 1,026, and 827 down-regulated gene expressions, respectively (Figure 3). A total of 739 altered gene entities are shared between ESBL019 Transition and ESBL019 Filamented with 585 up-regulated and 157 down-regulated entities, and 1,239 gene entities were shared between ESBL019 Filamented and ESBL019 Reverted with 914 up-regulated and 325 down-regulated entities. In addition, 381 shared gene entities were differentially expressed in all three groups (Figure 3).

### Gene ontology and pathway analysis

Gene ontology (GO) analysis was performed on significantly altered gene entities compared to ESBL019 Coliform expression. In total, 14 (ESBL019 Transition), 9 (ESBL019 Filamented) and 4 (ESBL019 Reverted) gene ontologies were uniquely enriched (Table S2). Single experiment pathway analysis (SEA) (*p* < 0.05) was performed in order to determine significantly affected cellular pathways. In total, 33 pathways were altered, of which 30 pathways were related to metabolism and the rest to enterobactin biosynthesis and polymyxin resistance (Table S3).

### Virulence factors in the different morphological states

Next, a more detailed analysis of the alterations in expression patterns of virulence associated genes of ESBL019 was conducted. The compiled list of virulence factor associated genes, 305 entities in total, was used to filter out significantly altered entities associated with virulence in the different groups. In total, 199 altered gene entities were identified with association to virulence. These 199 altered gene entities were grouped into 10 different functional groups; Adhesion, Growth, LPS synthesis, Metabolism, Motility, Protection, Siderophore, Toxins, Secretion and Transcription regulation. Of the 199 genes, 19 genes had significantly altered expressions in all three morphological states compared to the original ESBL019 Coliform (Table 1A). The ESBL019 Transition state showed altered expression of 33 virulence associated genes (Tables 1A,B). The ESBL019 Filamented state showed the single largest alteration in virulence associated gene expression. A total of 149 altered expression in genes was observed in the ESBL019 Filamented state (Tables 1A,B). The ESBL019 Reverted state showed altered expression of 92 virulence associated genes (Tables 1A,B).

**Table 1A:** Shared virulence factors associated genes present among significantly altered entities in ESBL019 Transition, ESBL019 Filamented and ESBL019 Reverted compared to ESBL019 Coliform.

**Function**	**Gene**	**ESBL019 Coliform**	**Fold change vs. ESBL019 coliform**			**Description**
		**Normalized entity fluorescence intensity signals**	**ESBL019 transition**	**ESBL019 filamentous**	**ESBL019 reverted**	
Adhesin	papG	5	2.4	3.0	2.7	PapG protein
	papF	5	2.8	4.2	2.2	PapF protein
Growth	carB	7,750	−2.5	−6.0	−2.7	Carbamoyl-phosphate synthase large subunit
LPS synthesis	waaV	4	3.0	4.3	2.3	Putative beta1.3-glucosyltransferase
	rfbC	4	2.9	6.5	2.7	dTDP-6-deoxy-D-glucose-3.5 epimerase
Metabolism	mglB	2,552	−3.7	−10.0	3.5	D-galactose-binding periplasmic protein precursor
Motility	fliC	5	2.4	3.8	2.2	Flagellar biosynthesis flagellin. filament structural protein
Protection	tcpC	6	−4.2	−4.1	−2.1	TCP pilus biosynthesis protein tcpC
	ompT	5,577	2.5	−2.7	−5.2	Protease VII precursor
	wcaE	13	3.6	31.7	3.2	Putative colanic acid biosynthesis glycosyl transferase
	ibpA	155	7.1	37.4	4.3	Heat shock protein
	ibpB	124	6.0	56.6	12.0	Heat shock protein
Siderophore	sitB	1,841	−4.5	−10.9	−2.8	SitB protein
	chuT	1,255	−7.0	−9.1	−2.3	Putative Periplasmic binding protein
	fepE	5,165	−2.4	−5.4	−2.9	Ferric enterobactin transport protein fepE
	fepA	2,007	−3.0	−3.9	−2.6	Ferrienterobactin receptor precursor
	iroN	5	2.6	3.5	2.9	Siderophore receptor IroN
	entF	6	2.4	3.7	2.0	ATP-dependent serine activating enzyme
Toxins	hlyC	7	2.8	3.7	2.1	Hemolysin C

**Table 1B:** Virulence factors associated genes present among significantly altered entities in ESBL019 Transition, ESBL019 Filamented and ESBL019 Reverted compared to ESBL019 Coliform.

**Function**	**Gene**	**ESBL019 coliform**	**Fold change vs. ESBL019 Coliform**			**Description**
		**Normalized entity fluorescence intensity signals**	**ESBL019 transition**	**ESBL019 filamentous**	**ESBL019 reverted**	
Protection	degP	6,718	2.1			Periplasmic serine protease Do; heat shock protein HtrA
Siderophore	shiF	344	−3.0			ShiF protein
	fepC	119	−2.1			ATP-binding component of ferric enterobactin transport
Toxins	hlyA	6	−2.6			Hemolysin A
Growth	carA	3,789	−2.2	−2.3		Carbamoyl-phosphate synthetase. glutamine
Metabolism	mglA	801	−4.5	−16.2		ATP-bind. comp. of methyl-galactoside transp. and galactose taxis
	dctA	3,224	−5.0	−8.0		Uptake of C4-dicarboxylic acids
Protection	ivy	1,292	3.3	4.4		orf. hypothetical protein
Siderophore	entE	763	−2.3	−5.7		2.3-dihydroxybenzoate-AMP ligase
	entC	708	−2.9	−4.3		isochorismate hydroxymutase 2. enterochelin biosynthesis
	iucA	641	−2.2	−2.9		IucA protein
	fepB	269	−2.2	−2.6		Ferric enterobactin
	iroB	10	2.0	2.7		Putative glucosyltransferase
	chuA	595	−2.0	−2.5		Outer membrane heme/hemoglobin receptor
Adhesin	papX	3,768		−5.5		PapX protein
	focX	6,009		−5.4		Putative Regulatory protein
	csgE	234		−2.5		Curli production assembly/transport component. 2nd curli operon
	flu	796		−2.4		Outer membrane fluffing protein. similar to adhesin
	ompA	145,147		−2.2		Outer membrane protein 3a
	sfaB	40		2.4		Putative F1C and S fimbrial switch Regulatory
	papI	20		2.6		PapI protein
	phoU	1,346		2.7		neg. reg. for pho regulon and putative enzyme in phosphate metab.
	focG	18		2.7		F1C minor fimbrial subunit protein G presursor
	focD	11		2.9		F1C fimbrial usher
	papH	12		3.1		PapH protein
	focC	14		3.1		F1C periplasmic chaperone
	focH	23		3.1		F1C Putative fimbrial adhesin precursor
	csgA	5		3.3		Curlin major subunit. coiled surface structures; cryptic
	focF	14		3.4		F1C minor fimbrial subunit F precursor
	papA	21		3.6		PapA protein
	papK	10		3.7		PapK protein
	papD	1,267		3.9		PapD protein
	papC	9		3.9		PapC protein
	papE	13		4.0		PapE protein
	fimE	351		5.5		Recombinase involved in phase variation; regulator for fimA
	fimB	40		6.8		Recombinase involved in phase variation; regulator for fimA
LPS synthesis	waaI	14		2.2		Putative LPS biosynthesis enzyme
	waaJ	3		2.2		Putative LPS biosynthesis enzyme
	waaL	39		2.5		Lipid A-core. surface polymer ligase
	waaA	105		2.7		3-deoxy-D-manno-octulosonic-acid transferase
	yibD	93		3.2		Putative regulator
	rfbA	23		3.2		Glucose-1-phosphate thymidylyltransferase
	waaY	11		3.8		Putative LPS biosynthesis protein
Metabolism	dppA	8,306		−8.6		Dipeptide transport protein
	narH	262		−4.1		Nitrate reductase 1. beta subunit
	narG	214		−3.9		Nitrate reductase 1. alpha subunit
	fruR	3,876		−3.3		Transcriptional repressor of fru operon and others
	deoC	1,595		−2.6		2-deoxyribose-5-phosphate aldolase
	malX	201		−2.3		PTS system. maltose and glucose-specific II ABC
	lysR	81		2.1		Positive regulator for lys
	ycjV	10		2.5		Putative ATP-binding component of a transport system
	gatC	88		2.6		PTS system galactitol-specific enzyme IIC
	btuB	147		2.8		Outer memb. Rec. for transp. of vit B12. E colicins and bact.phage
	wbdP	3		3.0		Glycosyl transferase
	ycfQ	354		3.2		orf. hypothetical protein
	yedL	5		3.6		orf. hypothetical protein
	ycjM	69		8.6		Putative sucrose phosphorylase
Motility	flhD	638		−5.6		reg. of flag. biosynth. acting on class 2 operons; transcrip. Init. factor
	flhC	365		−3.5		reg. of flag. Biosynth. acting on class 2 operons; transcrip. Init. factor
	fliY	3,253		−2.5		Putative periplasmic binding transport protein
	flgM	281		−2.1		Anti-FliA
	fliG	22		2.1		flag. biosynth. component of motor switching and energizing
	fliZ	156		2.2		orf. hypothetical protein
	flhE	8		2.2		Flagellar protein
	flgA	134		2.4		Flagellar biosynthesis; assembly of basal-body periplasmic P ring
	fliD	17		2.5		Flag. biosynth; filament capping protein; enables filament assembly
	fliI	39		2.5		Flagellum-specific ATP synthase
	fliK	168		3.4		Flagellar hook-length control protein
Protection	ompF	45,769		−7.3		Outer membrane protein 1a
	kdpE	132		2.1		Regulator of kdp operon
	sbmA	231		2.1		Sensitivity to microcin B17. possibly envelop protein
	gfcC	9		2.3		orf. hypothetical protein
	sdiA	295		2.5		Regulatory protein sdiA
	etk	23		2.7		orf. hypothetical protein
	etp	26		2.7		Putative phosphatase
	yadK	10		2.7		Protein yadK
	ybcL	84		3.0		Protein ybcL precursor
	gfcE	10		3.1		Putative function in exopolysaccharide production
	gfcD	553		3.9		orf. hypothetical protein
	ycbQ	8		4.3		Putative fimbrial-like protein
	gfcA	278		4.6		orf. hypothetical protein
	sulA	2,130		5.4		Suppressor of lon; inhibits cell division and ftsZ ring formation
	recA	1,662		6.0		DNA strand exchange and renaturation. DNA-dep ATPase
	lexA	489		6.3		Regulator for SOS
	bhsA	241		8.8		orf. hypothetical protein
Secretion	tir	39		2.2		Putative translocated intimin receptor protein
	escJ	15		2.5		escJ
	escN	15		2.8		escN
	espB	8		2.8		Secreted protein EspB
	sepQ	3		3.1		sepQ
	sepZ	11		3.3		sepZ
	eae	20		3.4		Intimin adherence protein
	escU	49		3.6		escU
Siderophore	entB	345		−3.6		2.3-dihydro-2.3-dihydroxybenzoate synthetase. isochroismatase
	entF	6		−3.5		Enterobactin synthetase component F
	iucC	867		−3.5		IucC protein
	entA	262		−3.4		2.3-dihydro-2.3-dihydroxybenzoate dehydrogenase
	chuS	655		−2.8		Putative heme/hemoglobin transport protein
	fepG	967		−2.7		Ferric enterobactin transport protein
	iucB	365		−2.6		IucB protein
	feoB	937		−2.5		Ferrous iron transport protein B
	iroE	10		3.7		IroE protein
Toxins	stx1A	22		2.3		Shiga-like toxin 1 subunit A encoded within
	hlyE	135		2.8		Hemolysin E
	stx2B	6		3.6		Shiga-like toxin II B subunit
	stx1B	12		4.3		Shiga-like toxin 1 subunit B
Transcription regulation	rfaH	384		2.1		Transcription act. of lipopolysaccharide core. F pilin. and haemolysin
Adhesin	fimC	26		2.1	3.5	Periplasmic chaperone. required for type 1 fimbriae
	fimI	39		2.1	3.4	Fimbrial protein
	fimH	36		2.1	3.6	Minor fimbrial subunit. D-mannose specific adhesin
	fimD	25		2.2	2.9	Outer membrane protein; export and assembly of type 1 fimbriae
	fimA	126		2.0	2.3	Major type 1 subunit fimbrin
	csgC	19		2.6	2.0	Putative curli production protein
	fimG	27		2.8	2.5	Fimbrial morphology
	fimF	8		3.6	3.3	FimF protein precursor
LPS synthesis	yijP	4,354		−2.3	−3.8	Protein yijP
Metabolism	glnA	8,617		−86.0	−15.1	Glutamine synthetase
	ilvD	3,921		−11.3	−8.2	Dihydroxy-acid dehydratase
	serA	2,494		−9.6	−3.7	D-3-phosphoglycerate dehydrogenase
	tdcE	123		−3.6	3.7	Keto-acid formate acetyltransferase
	trpB	573		−3.0	−2.5	Tryptophan synthase. beta protein
	kbaZ	9		2.3	16.0	Putative tagatose 6-phosphate kinase 2
	pitB	26		2.8	3.2	Low-affinity phosphate transport
	aer	29		2.8	3.3	Aerotaxis sensor receptor. flavoprotein
	trxC	276		3.5	3.3	Thioredoxin 2
Motility	fliJ	13		3.4	2.3	Flagellar fliJ protein
Protection	ompW	4,714		−9.1	2.6	Outer membrane protein W precursor
	matB	614		−2.5	2.2	orf. hypothetical protein
	evgS	526		−2.2	−2.9	Putative sensor for regulator EvgA
	ECs0850	5		4.6	2.2	Hypothetical protein
	manB	11		8.5	2.3	Phosphomannomutase
Siderophore	yjjQ	68		2.1	2.4	putative regulator
Transcription regulation	rpoB	12,927		−2.3	−4.5	RNA polymerase. beta subunit
	rpoA	68,440		−2.3	−3.7	RNA polymerase. alpha subunit
Adhesin	ppdD	21			2.3	Prelipin peptidase dependent protein
LPS synthesis	rfaS	12,88			−4.3	Lipopolysaccharide core biosynthesis
	rfaP	3,909			−3.8	LPS core biosynthesis; phosphorylation of core heptose
	rfaZ	2,091			−3.0	Lipopolysaccharide core biosynthesis
	rfaY	1,690			−2.5	Lipopolysaccharide core biosynthesis
	waaU	999			−2.1	Probably hexose transferase; lipopolysaccharide core biosynthesis
	rfaG	1,009			−2.0	Lipopolysaccharide core biosynthesis protein rfaG
Metabolism	eda	3,246			−3.1	2-keto-3-deoxygluc 6-phos aldo and 2-keto-4-hydroxyglut aldo
	tktA	2,823			−2.2	Transketolase 1 isozyme
	dsbA	27,226			−2.1	Protein disulfide isomerase I. essential for cytochrome c synthesis
	prfB	3,054			−2.0	Peptide chain release factor 2
	gnsA	3,461			−2.0	Predicted regulator of phosphatidylethanolamine synthesis
	yjhS	37			2.1	orf. hypothetical protein
	agaA	113			2.5	Putative N-acetylgalactosamine-6-phosphate deacetylase
	agaC	42			2.7	PTS system N-acetylgalactosamine-specific IIC component 1
	dctA	3,224			3.2	Uptake of C4-dicarboxylic acids
	astA	93			3.6	orf. hypothetical protein
	lacZ	20			3.7	Beta-galactosidase
	gatZ	18			4.1	Putative tagatose 6-phosphate kinase 1
	gatB	15			5.0	Galactitol-specific enzyme IIB of phosphotransferase system
	agaS	72			5.3	Putative tagatose-6-phosphate aldose/ketose isomerase
	agaB	32			7.0	PTS system. cytopl. N-acetylgalactosamine-spec IIB component 1
	ulaB	27			7.2	Putative transport protein
	ulaC	86			8.6	Putative PTS system enzyme II A component
	ulaA	76			11.2	orf. hypothetical protein
	agaW	40			18.1	PTS system N-acetylgalactosameine-specific IIC component 2
	agaV	19			18.9	PTS system. cytopl. N-acetylgalactosamine-spec IIB component 2
	kbaY	19			28.0	Tagatose-bisphosphate aldolase 2
Motility	fliT	31			2.1	Flagellar biosynthesis; repressor of class 3a and 3b operons
	flgJ	29			2.1	Flagellar biosynthesis
	fliO	11			2.1	Flagellar protein fliO
	fliL	23			2.2	Flagellar biosynthesis
	flgH	20			2.2	Flagellar biosynthesis. basal-body outer-membrane L
	flhB	38			2.3	Putative part of export apparatus for flagellar proteins
	flgB	24			2.3	Flagellar biosynthesis. cell-proximal portion of basal-body rod
	fliE	37			2.7	Flagellar biosynthesis; basal-body component
	fliF	27			2.8	Flagellar biosynthesis; basal-body MS
	lfhA	8			3.1	Flagellar biosynthesis
	fliP	11			3.3	Flagellar biosynthetic protein fliP precursor
	fliQ	21			4.9	Flagellar biosynthesis
Protection	kpsC	986			−3.4	KpsC protein
	bamA	6,961			−3.3	orf. hypothetical protein
	evgS	526			−2.9	putative sensor for regulator EvgA
	degP	6,718			−2.6	Periplasmic serine protease Do; heat shock protein HtrA
	kpsM	3,290			−2.2	KpsM protein
Transcription regulation	fis	61,722			−3.9	Site-specific DNA inversion stimulation factor; DNA-binding protein

In order to confirm the microarray results, real-time qPCR was carried out on seven genes belonging to the functional categories Adhesion (*FimAH*), Protection (*ibpAB*), and Siderophore (*chuAT, sitB*) that were significantly altered compared to ESBL019 Coliform. In agreement with the microarray, a significant upregulation of *FimAH* was observed in ESBL019 Filamented and ESBL019 Reverted state compared to ESBL019 Coliform (Table 2). In addition, *ibpAB* was observed to be significant upregulated in all the three different states compared to ESBL019 Coliform, as shown by the microarray data. We also found that *chuAT, sitB* were significantly downregulated in ESBL019 Transition and ESBL019 Filamented state compared to ESBL019 Coliform (Table 2). However, this was not observed to be true for the ESBL019 Reverted state.

**Table 2:** Quantitative real-time PCR data for ESBL019 Transition, ESBL019 Filamented and ESBL019 Reverted compared to ESBL019 Coliform.

**Gene**		**Fold change vs. ESBL019 coliform**		**Description**
	**ESBL019 transition**	**ESBL019 filamentous**	**ESBL019 reverted**	
FimA	−1.2 ± 0.86^a^	15 ± 3.1^a^	8.5 ± 2.1^a^	Major type 1 subunit fimbrin
FimH	−1.1 ± 0.74^a^	10 ± 1.8^a^	11 ± 2.3^a^	Minor fimbrial subunit. D-mannose specific adhesin
ibpA	5.3 ± 0.90^a^	230 ± 49^a^	22 ± 3.7^a^	Heat shock protein
ibpB	7.2 ± 1.5^a^	330 ± 80^a^	130 ± 33^a^	Heat shock protein
chuA	−6.3 ± 1.2^a^	−1.6 ± 0.1^a^	0.90 ± 1.0	Putative Periplasmic binding protein
chuT	−15 ± 3.2^a^	−4.4 ± 1.5^a^	1.1 ± 1.0	Outer membrane heme/hemoglobin receptor
sitB	−11 ± 1.6^a^	−5.4 ± 1.6^a^	0.71 ± 0.82	SitB protein

*n = 3–4*.

a *Significantly altered genes compared to ESBL019 Coliform*.

### Fimbriae agglutination

**Figure 4:**
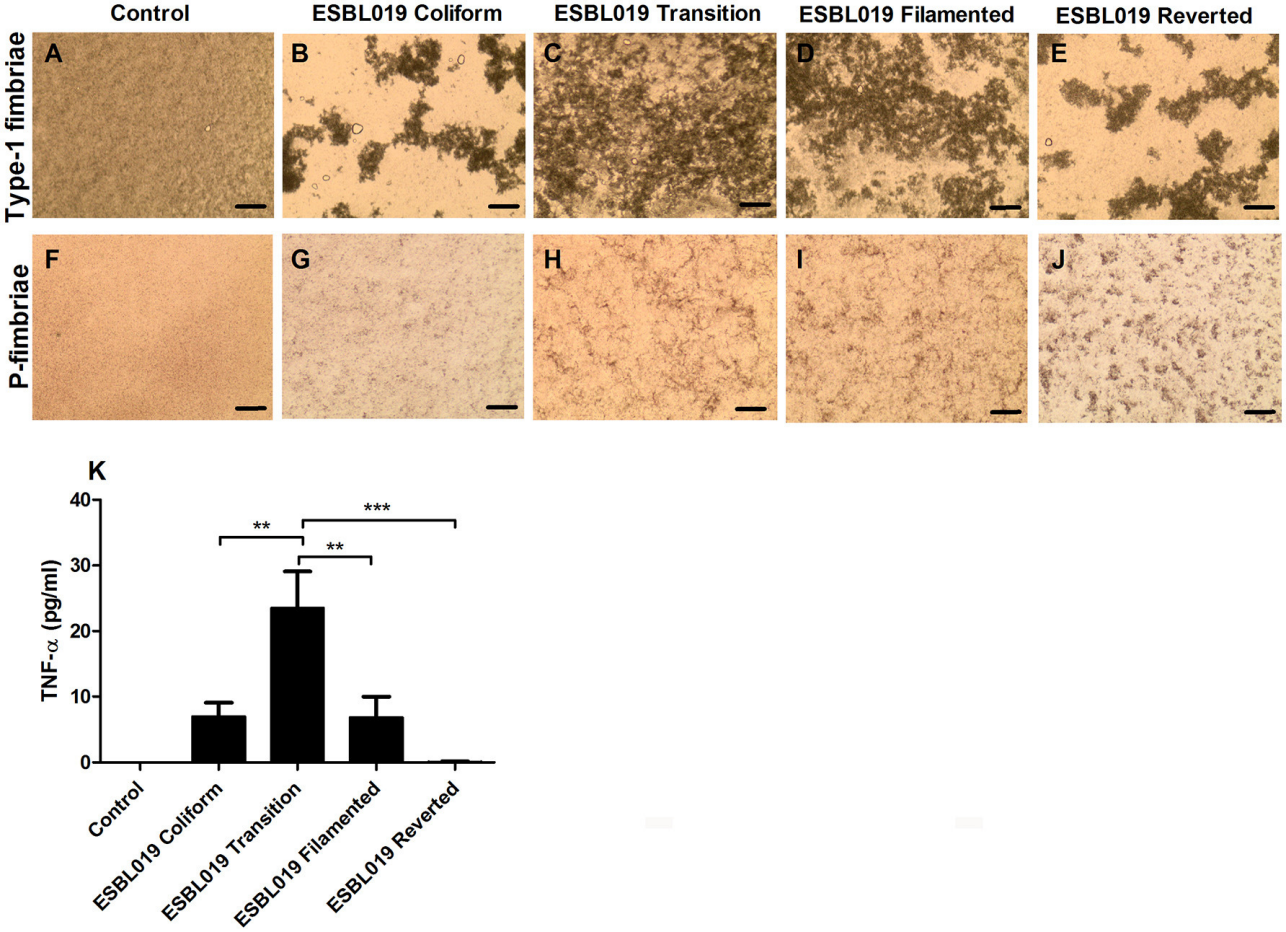
Fimbriae aggregation and TNF-α release from HBEP cells infected with the different morphologies of ESBL019. Type-1 fimbriae functionality was assessed by yeast agglutination without **(A)** or with ESBL019 Coliform **(B)**, ESBL019 Transition **(C)**, ESBL019 Filamented **(D)** and ESBL019 Reverted **(E)** using light microscopy. P-fimbriae functionality was assessed by P-positive human erythrocyte agglutination without **(F)** or with ESBL019 Coliform **(G)**, ESBL019 Transition **(H)**, ESBL019 Filamented **(I)**, and ESBL019 Reverted **(J)** using light microscopy. Scale bare: 200 μm. TNF-α release from HBEP cells stimulated with ESBL019 Coliform, ESBL019 Transition, ESBL019 Filamented and ESBL019 Reverted (MOI10) for 4 h in the presence (Transition, Filamented) or absence (Coliform and Reverted) of ceftibuten **(K)**. Data are presented as mean ± SEM of *n* = 4 independent experiments. Asterisks denote statistical significance ^**^p < 0.01, ^***^p < 0.001.

We proceeded with evaluating the phenotypical presence and functionality of the type-1 fimbriae and P-fimbriae in the different morphological states of ESBL019. Yeast agglutination confirmed that all the morphological states of ESBL019 expressed functional type-1 fimbriae (Figures 4B–E) compared to only yeast (Figure 4A). The aggregation pattern between the rod shaped (ESBL019 Coliform and ESBL019 Reverted) and the elongated ESBL019 (ESBL019 Transition and ESBL019 Filamented) morphologies were strikingly different. The yeast was bound along the elongated bacteria and gave a more uniform aggregation (Figures 4C,D), while the rod shaped bacteria formed large isolated yeast aggregates (Figures 4B,E). Human Erythrocytes of blood group P were used to evaluate the P-fimbriae expression. All the morphological states of ESBL019 expressed functional P-fimbriae (Figures 4G–J) compared to only erythrocytes (Figure 4F). We also observed an increased aggregation by the ESBL019 Transition (Figure 4H), ESBL019 Filamented (Figure 4I) and ESBL019 Reverted (Figure 4J) states compared to ESBL019 Coliform (Figure 4G).

### Epithelial TNF-α release evoked by the different morphological states

Primary human bladder epithelial cells were stimulated with the different morphological states of ESBL019 to determine their ability to induce the pro-inflammatory cytokine TNF-α. The unstimulated control cells showed no basal release of TNF-α (Figure 4K). ESBL019 Coliform and ESBL Filamented were able to induce an increased release of TNF-α compared to the control. ESBL019 Transition induced a significant higher release of TNF-α compared to control (*p* < 0.001), ESBL019 Coliform (*p* < 0.01), ESBL019 Filamented (*p* < 0.01) and ESBL019 Reverted (*p* < 0.001). In contrast to the other morphological states of ESBL019, ESBL019 Reverted did not induce any release of TNF-α from the bladder epithelial cells. Taken together, the different morphological states of ESBL019 induce a diverse TNF-α release from bladder epithelial cells.

## Discussion

Global gene expression profiling of the different morphological states of ESBL019 demonstrated a significant transcriptional alteration of a large number of genes. In the ESBL019 Transition state, close to 7% of the entities on the array were altered, 32% for ESBL019 Filamented and 19% for ESBL019 Reverted. Thus, it appears that the morphological plasticity of UPEC is associated with an extensive flux in the transcriptome of the bacteria and especially in the filamented state. To the best of our knowledge, this is the first study that investigates changes in global gene expression patterns and in virulence factor associated genes in ESBL-producing UPEC during the morphologic transitions induced by ineffective antibiotics. Importantly, all experiments were conducted in the presence of human primary bladder epithelial cells to mimic the *in vivo* host-pathogen interactions in a physiologically relevant manner.

The majority of the identified changes in gene ontologies and pathways were associated with metabolic functions. Many metabolic functions are not specific for pathogens and their role in infection fitness remains therefore more or less unexplored (Subashchandrabose and Mobley, 2015). In the ESBL019 Transition state, we observed that genes associated with the tricarboxylic acid cycle (TCA) and glucogenic amino acid (arginine, glutamate, glutamine, and proline) metabolism were in majority downregulated. We also found that the gene *dctA*, which encodes for the C4-dicarboxylate transporter that is responsible for the uptake of fumarate, succinate and malate was downregulated. Previous studies have shown that gluconeogenesis and the TCA cycle, but not Entner-Doudoroff, glycolysis or pentose-phosphate pathways are important for the fitness of UPEC during *in vivo* experimental UTI (Alteri et al., 2009). Genes associated with amino acid metabolism, such as *dctA, serA* (L-serine biosynthesis), *ilvD* (isoleucine and valine biosynthesis), *trpB* (tryptophan synthase beta), *glnA* (catalyzing conversion of glutamate to glutamine) and *dppA* (dipeptide transporter) were observed to be downregulated in the ESBL019 Filamented state. It has been shown that UPEC grown in urine utilizes primarily peptides and amino acids as carbon sources instead of glucose (Alteri et al., 2009). We also observed that several sugar transporter associated genes such as *gatC* and *ycjV* were upregulated in the filamented state, which could lead to an increased acquisition of carbon sources. In the ESBL019 Reverted state, glucose and xylose degradation, glycolysis, Entner-Doudoroff, NAD biosynthesis and gluconeogenesis pathways were downregulated. We also found, in accordance with the filamented state, that *glnA, ilvD*, and *serA* were downregulated. In addition, an increase in genes associated with replenishing TCA cycle intermediates (*dctA* and *astA*), sugar transporters (*agaABCVW*), L-ascorbate transport (*ulaABC*) and degradation of other carbon sources like lactose (l*acZ*) was observed. It has recently been shown that beta-galactosidase (l*acZ*) may play an important role in intracellular UPEC growth and IBC maturation (Conover et al., 2016). An interesting finding in the ESBL019 Reverted state was the downregulation of genes associated with transcription, translation, ribosome biosynthesis and tRNA charging. Taken together, these data demonstrates that all three morphological states of UPEC are associated with a decreased energy-yielding cell metabolism, suggesting that survival rather than growth is prioritized. Knowledge of these metabolic shifts could contribute to finding new therapeutic targets to limit the bacteria's ability to acquire alternative carbon sources.

Metals such as iron are essential for bacterial life and the low concentration of soluble iron in the urinary tract is regarded as a limiting factor for UPEC growth (Roos et al., 2006). Consequently, it has been shown that iron acquisition is important for UPEC survival and pathogenicity (Subashchandrabose and Mobley, 2015). UPEC can acquire iron through several mechanisms such as siderophores (ferric iron chelators), ferrous iron transporters and heme through outer membrane receptors (Cho et al., 2008). We observed that the iron acquisition systems in all the morphological states of UPEC changed significantly. Genes involved in the enterobactin system (*entABF, fepABE*), heme system (*chuST*) and ferrous iron uptake (*sitB*) were downregulated in all the three morphological states. However, genes in the aerobactin system (*iucAC*) were downregulated in the ESBL019 Transition and ESBL019 Filamented state but not in the ESBL019 Reverted state. The salmochelin siderophore system (*iroEN*) was upregulated in all the three morphological states. qPCR analysis confirmed that *chuAT* and *sitB* were significantly downregulated in ESBL019 Transition and ESBL019 Filamented state, but not in the ESBL019 Reverted state compared to ESBL019 Coliform. The iron acquisition systems in UPEC have been demonstrated to be redundant, but individual systems have been shown to play key roles in fitness and pathogenicity in different regions of the urinary tract (Garcia et al., 2011). Aerobactin receptors have been shown to significantly contribute to *in vivo* fitness and they are critical for UPEC virulence (Garcia et al., 2011). It was recently shown that the virulence associated with iron acquisition could be partially due to modulation of amino acid metabolism (Su et al., 2016). Taken together, these data show that the iron acquisition systems in UPEC are altered during morphological shifts, mainly downregulated, but the redundancy in the systems may contribute to preserving *in vivo* fitness. In addition, the lack of change in the aerobactin system, one of the key siderophores system in uropathogenicity, may indicate that the reverted morphology maintains virulence in the life cycle of UPEC.

During colonization of the urinary tract, UPEC utilizes different adhesion molecules (like fimbriae) to adhere and invade the epithelial cells in order to persist in the urinary tract (Subashchandrabose and Mobley, 2015). The P-fimbriae encoding genes (*pap*) were upregulated in all the three different morphological states of ESBL019 and we also observed an increased phenotypic expression by agglutination in all the different morphological states compared to ESBL019 Coliform. The P-fimbriae is associated with the pathogenicity of ascending UTI and pyelonephritis (Frendeus et al., 2001). The type-1 fimbriae associated genes (*fim*), which play an important role in adhesion, invasion, IBC, and biofilm formation during cystitis (Flores-Mireles et al., 2015) were upregulated in ESBL019 Filamented and ESBL019 Reverted, but no changes were observed in ESBL019 Transition state. Consistent with the microarray data, *fimAH* were markedly upregulated in the Filamented and Reverted state based on qPCR data. We confirmed phenotypically functional type-1 fimbriae in all the different states by yeast agglutination assays. This indicates that the morphological plasticity of UPEC does not compromise on adhesion factors important for colonizing the urinary tract. We hypothesize that the observed upregulation of P-and type-1 fimbriae associated genes is a mechanism to persist in the urinary tract especially after the bacteria emerge from the IBC in a filamented form. Taken together, our results show that all UPEC morphologies maintain fimbrial expression, which could promote persistence and colonization of the urinary tract.

Ascendance from the bladder to the kidneys is an important virulence trait of UPEC associated with motility and flagella expression (Pichon et al., 2009). Studies of morphological plasticity of ESBL019 showed only one significantly upregulated flagellin gene in the ESBL019 Transition state (*fliC*), but many flagellin genes were both up-and downregulated in the ESBL019 Filamented state. In the ESBL019 Reverted state, all the significantly altered genes were upregulated. These results indicate that morphological plasticity is associated with altered flagellin gene expression, but additional functional experiments are needed to assess the effects on motility.

We and others have shown that filamentation of *E. coli* is associated with a rapid production and release of endotoxins (Gould and MacKenzie, 1997; Demirel et al., 2015). Genes associated with LPS biosynthesis were almost unchanged in the ESBL019 Transition state, but all LPS genes were upregulated in the ESBL019 Filamented state. However, the majority of the LPS associated genes were downregulated in the ESBL019 Reverted state. The modulation of LPS synthesis during the morphological shifts could be a compensation mechanism to replenish LPS on the surface of ESBL019, as increased shedding of LPS was observed during the transition state (Demirel et al., 2015). This modulation of LPS could play a part in the pathogenicity of UPEC as LPS modulates pro-inflammatory responses (Bussolati et al., 2002).

The morphological plasticity of ESBL019 showed an alteration in many genes associated with bacterial protection. Genes like *tcpC*, which is associated with inhibition of pro-inflammatory responses (Yadav et al., 2010), was downregulated in all three states. We also observed that the gene encoding for the outer membrane protein *ompT*, which is involved in the resistance of different antimicrobial peptides (Brannon et al., 2013), was upregulated in the transition state, but downregulated in the two other states. The gene encoding ompF, which is a porin for antibiotics, was downregulated in the filamented state and this has been associated with increased antibiotic resistance (Kishii and Takei, 2009). OmpW, which is required for UPECs resistance against phagocytosis (Wu et al., 2013) was downregulated in the filamented state, but upregulated in the reverted state. We have previously shown that filamented UPEC can be successfully phagocytized by neutrophils (Demirel et al., 2015). Related to neutrophil immunity, the *ybcL* gene proposed to inhibit neutrophil migration was upregulated in the ESBL019 Filamented state (Lau et al., 2012). The gene ontology SOS response was enriched in the ESBL019 Filamented state and the associated genes *recA, lexA*, and *sulA* were upregulated. The SOS system is a response to DNA damage and inhibits cell division and septa formation in order to repair the DNA damage (Justice et al., 2006). *sulA* has been shown to mediate UPEC filamentation and this filamentation is associated with increased genetic variability and point mutations that can lead to antibiotic resistance (Bos et al., 2015). The filamented form also showed an upregulation of genes (*tir, esc*, and *sep*) encoding the Type 3 secretion system (T3SS), which is used by bacteria to inject effectors directly into host cell (Subashchandrabose and Mobley, 2015). T3SS genes are traditionally not found in UPEC, but they have recently been reported to be found in some UPEC isolates (Subashchandrabose et al., 2013), although the role during UTI remains to be determined.

We continued with analyzing the TNF-α production from primary bladder epithelial cells induced by the different morphological states. This was done in order to evaluate the biological relevance of the microarray data. The ESBL019 Transition state induced a significant increase in TNF-α production compared to the control and the other morphologies. We have previously shown that the transition state is associated with increased LPS and ATP release, both known to induce pro-inflammatory cytokines from uroepithelial cells (Demirel et al., 2015). The reverted state did not induce TNF-α production and this may give the reverted UPEC an advantage in colonizing the bladder as host cytokine responses are essential for attracting neutrophils (Smart and Casale, 1994). However, it remains to be investigated if the lack of immune activation by the reverted state is due to an inability to induce an immune response or to an active immune suppression. Our agglutination assays showed no reduced fimbriae function to support that the reverted state is unable to induce an immune response. However, several interesting findings, like the downregulation of LPS associated genes in the reverted state, remains to be evaluated.

Together, these results provide insight into the global gene expression patterns of the different morphological states of ESBL019 induced by an ineffective antibiotic. The decreased energy-yielding metabolism and up-regulated adhesion, suggest that survival and persistence in the bladder is priority rather than growth during the morphological plasticity of ESBL-producing UPEC. These findings are also strengthened by the fact that the reverted state did not induce TNF-α release, which gives the bacteria an advantage in colonizing the bladder. Understanding how ineffective antibiotic treatments affect the virulence and metabolism of ESBL-producing UPEC can promote the identification of new therapeutic targets and new adjuvant combination treatments to reduce the prevalence of multiresistant bacteria.

## Author contributions

ID, RK and KP design the study. ID and RK conducted the experiments. ID, RK, IR, UP, and KP analyzed the data. ID, RK, IR, UP, and KP drafted the article. All authors read and approved the final manuscript.

### Conflict of interest statement

The authors declare that the research was conducted in the absence of any commercial or financial relationships that could be construed as a potential conflict of interest. The reviewer JM and handling Editor declared their shared affiliation, and the handling Editor states that the process nevertheless met the standards of a fair and objective review.
