# Supplementary material for: Transcriptional Alterations of Virulence-Associated Genes in Extended Spectrum Beta-Lactamase (ESBL)-Producing Uropathogenic Escherichia coli during Morphologic Transitions Induced by Ineffective Antibiotics
Source: Front Microbiol. 2017 Jun 13;8:1058. doi: 10.3389/fmicb.2017.01058 (PMC5468405; doi:10.3389/fmicb.2017.01058)
Supplement: Supplementary file 1 [file Table1.DOCX]

**Supplemental Table 1*.* Primers used for quantitative real-time PCR.**

| **Gene symbol** | **Oligonucleotide sequences (5´-3´)** | **References** |
| --- | --- | --- |
| FimA | *F:* CTGGCAATTGTTGTTCTGTCGGCT  *R:* ACGGTTTGATCAACAGAGCCTGC | (Berry et al., 2009) |
| *FimH* | *F:* GTGCCAATTCCTCTTACCGTT  *R:* TGGAATAATCGTACCGTTGCG | (Untergasser et al., 2012) |
| *ibpA* | *F:* AGAGCGCACCTATCTGTACC  *R:* AGTTGATTTCGATACGGCGC | (Untergasser et al., 2012) |
| *ibpB* | *F:* ACCACTACCGCATTACCCTT  *R:* TTGCGCCAGAGACTTCCATA | (Untergasser et al., 2012) |
| *chuA* | *F:* AAGGCGTTGCCCAATACCAGAGTA  *R:* TATTCCGATCGCTCACAGTGGCTT | (Berry et al., 2009) |
| *chuT* | *F:* AAACAACTCAGCAGCGAAGG  *R:* GGTTGATCTGTGTCACCAGC | (Untergasser et al., 2012) |
| *sitB* | *F:* CAATCTGCAGGCATTGTCGT  *R:* CTGGTCAGACGCACAAATCC | (Untergasser et al., 2012) |
| *gapA* | *F: AAGTTGGTGTTGACGTTG*  *R: AGCGCCTTTAACGAACATCG* | (Alteri and Mobley, 2007) |

Alteri, C.J., and Mobley, H.L. (2007). Quantitative profile of the uropathogenic Escherichia coli outer membrane proteome during growth in human urine. *Infect Immun* 75**,** 2679-2688.

Berry, R.E., Klumpp, D.J., and Schaeffer, A.J. (2009). Urothelial Cultures Support Intracellular Bacterial Community Formation by Uropathogenic Escherichia coli. *Infection and Immunity* 77**,** 2762-2772.

Untergasser, A., Cutcutache, I., Koressaar, T., Ye, J., Faircloth, B.C., Remm, M., and Rozen, S.G. (2012). Primer3--new capabilities and interfaces. *Nucleic Acids Res* 40**,** e115.
