# Supplementary material for: Transcriptional Alterations of Virulence-Associated Genes in Extended Spectrum Beta-Lactamase (ESBL)-Producing Uropathogenic Escherichia coli during Morphologic Transitions Induced by Ineffective Antibiotics
Source: Front Microbiol. 2017 Jun 13;8:1058. doi: 10.3389/fmicb.2017.01058 (PMC5468405; doi:10.3389/fmicb.2017.01058)
Supplement: Supplementary file 2 [file Table2.DOCX]

| **Supplemental Table 2.** Enriched gene ontologies among significantly altered entities in ESBL019 Transition. ESBL019 Filamented and ESBL019 Reverted compared to ESBL019 Coliform. | | | | | | | |
| --- | --- | --- | --- | --- | --- | --- | --- |
|  | **Function** | **GO ID** | **GO Term** | **p-value** | **Counts in selection** | **Counts in total** | **Regulation up/down** |
| **ESBL019 Transition** | *Response to stimulus* | 6894 | response to abiotic stimulus | 0.007 | 18 | 71 | 14/4 |
|  |  | 6627 | response to temperature stimulus | 0.048 | 8 | 21 | 5/3 |
|  | *Metabolic process* | 4366 | tricarboxylic acid cycle | 0.007 | 9 | 20 | 1/8 |
|  |  | 4367 | citrate metabolic process | 0.007 | 9 | 20 | 1/8 |
|  |  | 4684 | arginine metabolic process | 0.007 | 10 | 24 | 0/10 |
|  |  | 6434 | glutamine family amino acid metabolic process | 0.007 | 16 | 52 | 0/16 |
|  |  | 11666 | carboxylic acid metabolic process | 0.007 | 53 | 347 | 12/41 |
|  |  | 20480 | oxoacid metabolic process | 0.007 | 53 | 347 | 12/41 |
|  |  | 32311 | tricarboxylic acid metabolic process | 0.007 | 9 | 20 | 1/8 |
|  |  | 21296 | small molecule biosynthetic process | 0.027 | 34 | 202 | 12/22 |
|  |  | 36525 | alpha-amino acid metabolic process | 0.027 | 28 | 154 | 7/21 |
|  |  | 6081 | cellular amino acid biosynthetic process | 0.041 | 28 | 158 | 7/21 |
|  |  | 6436 | aspartate family amino acid metabolic process | 0.049 | 12 | 44 | 6/6 |
|  |  | 4354 | organic acid metabolic process | 0.007 | 53 | 350 | 12/41 |
|  |  |  |  |  |  |  |  |
| **ESBL019 Filamented** | *Response to stimulus* | 6762 | SOS response | 0.016 | 19 | 24 | 19/0 |
|  |  | 14013 | cellular response to extracellular stimulus | 0.016 | 19 | 24 | 19/0 |
|  | *Metabolic process* | 4361 | generation of precursor metabolites and energy | 0.000 | 111 | 199 | 28/83 |
|  |  | 9220 | energy derivation by oxidation of organic compounds | 0.001 | 103 | 187 | 28/75 |
|  |  | 6431 | anaerobic respiration | 0.002 | 80 | 141 | 22/58 |
|  |  | 22162 | cellular respiration | 0.003 | 92 | 169 | 24/68 |
|  |  | 28315 | oxidation-reduction process | 0.009 | 116 | 227 | 36/80 |
|  |  | 6425 | electron carrier activity | 0.010 | 57 | 97 | 17/40 |
|  | *Other* | 5201 | cell communication | 0.016 | 19 | 24 | 19/0 |
|  |  |  |  |  |  |  |  |
| **ESBL019 Reverted** | *Biosynthesis* | 2711 | structural constituent of ribosome | 0.000 | 50 | 53 | 0/50 |
|  |  | 4603 | translation | 0.000 | 79 | 108 | 3/76 |
|  |  | 15101 | monocarboxylic acid metabolic process | 0.022 | 49 | 86 | 33/16 |
|  |  | 11477 | protein metabolic process | 0.022 | 142 | 309 | 42/100 |
