# Supplementary material for: Transcriptional Alterations of Virulence-Associated Genes in Extended Spectrum Beta-Lactamase (ESBL)-Producing Uropathogenic Escherichia coli during Morphologic Transitions Induced by Ineffective Antibiotics
Source: Front Microbiol. 2017 Jun 13;8:1058. doi: 10.3389/fmicb.2017.01058 (PMC5468405; doi:10.3389/fmicb.2017.01058)
Supplement: Supplementary file 3 [file Table3.DOCX]

| **Supplemental Table 3.** Single Experiment Pathway Analysis of significantly altered entities in ESBL019 Transition, ESBL019 Filamented and ESBL019 Reverted compared to ESBL019 Coliform. | | | | |
| --- | --- | --- | --- | --- |
| **Pathway** | **p-value** | **Matched Entities** | **Pathway Entities** | **Regulation up/down** |
| ***ESBL019 Transition*** |  |  |  |  |
| arginine biosynthesis I | 0.01 | 2 | 2 | 0/2 |
| superpathway of arginine and polyamine biosynthesis | 0.01 | 2 | 2 | 0/2 |
| polymyxin resistance | 0.02 | 2 | 3 | 0/2 |
| 4-amino-2-methyl-5-diphosphomethylpyrimidine biosynthesis | 0.09 | 1 | 4 | 1/0 |
| enterobactin biosynthesis | 0.09 | 1 | 1 | 0/1 |
| ornithine biosynthesis | 0.09 | 1 | 1 | 0/1 |
| L-lactaldehyde degradation | 0.09 | 1 | 1 | 0/1 |
| D-gluconate degradation | 0.09 | 1 | 1 | 1/0 |
| acetate conversion to acetyl-CoA | 0.09 | 1 | 5 | 0/1 |
| methylglyoxal degradation IV | 0.09 | 1 | 1 | 0/1 |
| 2,3-dihydroxybenzoate biosynthesis | 0.09 | 1 | 4 | 0/1 |
| glycine cleavage | 0.09 | 1 | 4 | 0/1 |
|  |  |  |  |  |
| ***ESBL019 Filamented*** |  |  |  |  |
| methylglyoxal degradation III | 0.04 | 4 | 6 | 1/3 |
| superpathway of methylglyoxal degradation | 0.03 | 6 | 7 | 1/5 |
|  |  |  |  |  |
| ***ESBL019 Reverted*** |  |  |  |  |
| superpathway of dTDP-glucose-derived O-antigen building blocks biosynthesis | 0.00 | 4 | 24 | 1/3 |
| tRNA charging | 0.02 | 5 | 7 | 0/5 |
| aspartate superpathway | 0.02 | 4 | 5 | 1/3 |
| superpathway of N-acetylneuraminate degradation | 0.04 | 4 | 6 | 1/3 |
| glucose and xylose degradation | 0.04 | 4 | 6 | 0/4 |
| superpathway of glycolysis and Entner-Doudoroff | 0.04 | 4 | 6 | 0/4 |
| gluconeogenesis I | 0.06 | 3 | 14 | 1/2 |
| threonine degradation I | 0.07 | 2 | 3 | 1/1 |
| D-galactonate degradation | 0.07 | 2 | 3 | 2/0 |
| NAD biosynthesis I | 0.07 | 2 | 4 | 0/2 |
| superpathway of acetate utilization and formation | 0.07 | 2 | 2 | 1/1 |
| histidine biosynthesis | 0.07 | 2 | 10 | 0/2 |
| L-ascorbate degradation I | 0.07 | 2 | 2 | 2/0 |
| dTDP-N-acetylthomosamine biosynthesis | 0.07 | 2 | 2 | 0/2 |
| superpathway of threonine metabolism | 0.07 | 2 | 3 | 1/1 |
| dTDP-L-rhamnose biosynthesis I | 0.07 | 2 | 6 | 1/1 |
| thiosulfate disproportionation III | 0.07 | 2 | 3 | 2/0 |
| superpathway of microbial D-galacturonate and D-glucuronate degradation | 0.09 | 5 | 21 | 5/0 |
| Lipid A-core biosynthesis | 0.09 | 5 | 10 | 0/5 |
